# Diagnostic relevance of osteocalcin and bone alkaline phosphatase as markers of bone turnover in Sickle Cell Anaemia: A Zaria study

**DOI:** 10.4314/ahs.v26i2.8

**Published:** 2026-06

**Authors:** Abdulfatai K Ogunkunle, Fatima A Mahmud, Murjanatu G Abubakar, Aminat B Dogara, Waliu T Adanlawo, Rasheed Yusuf, Ibrahim S Aliyu

**Affiliations:** 1 Department of Chemical Pathology, Ahmadu Bello University, Zaria; 2 Department of Chemical Pathology, Ahmadu Bello University Teaching Hospital, Zaria; 3 Department of Chemical Pathology, Federal University, Birnin-Kebbi & Federal Teaching Hospital, Birnin-Kebbi; 4 Department of Chemical Pathology, Kaduna State University and Barau Dikko Teaching Hospital, Kaduna; 5 Department of Chemical Pathology, Usumanu Danfodio University Teaching Hospital, Sokoto

**Keywords:** SCA, osteocalcin, BALP, bone turnover

## Abstract

**Background:**

Sickle cell anaemia (SCA) significantly contributes to skeletal morbidity in Nigeria, with bone complications like pain crises and avascular necrosis prevalent. Osteocalcin (OC) and bone-specific alkaline phosphatase (BALP) are potential markers of bone formation, yet their diagnostic utility in SCA is underexplored in African populations.

**Objective:**

To assess serum OC and BALP levels in Nigerian SCA patients during bone pain crises (BPC), steady state, and in healthy controls, evaluating their diagnostic relevance for bone turnover.

**Methods:**

This cross-sectional study at Ahmadu Bello University Teaching Hospital, Zaria, enrolled 180 adults (60 SCA in BPC, 60 steady state, 60 HbAA controls). Serum OC and BALP were measured via ELISA. Data were analyzed using Kruskal-Wallis tests, Spearman's correlation, and ROC curves (p≤0.05).

**Results:**

Median OC and BALP levels were significantly higher in SCA BPC (26.53 ng/mL, 23.51 µg/L) and steady state (24.40 ng/mL, 14.42 µg/L) compared to controls (16.07 ng/mL, 9.95 µg/L; p=0.022, p<0.001). BALP showed superior diagnostic accuracy (AUC=0.789, sensitivity=98.3%) compared to OC (AUC=0.592). OC correlated with BMI (p=0.020), but not age or gender.

**Conclusion:**

BALP is a more reliable marker than OC for detecting bone turnover alterations in SCA, particularly during BPC, supporting its use in resource-limited settings.

## Introduction

Sickle cell anaemia (SCA) remains a major public health problem in sub-Saharan Africa, and Nigeria in particular carries the greatest burden worldwide: an estimated 2–3% of the population is affected, and roughly 150,000 babies are born with the disease each year, such that chronic complications of SCA (including skeletal morbidity) contribute substantially to morbidity and early mortality.^1^,^2^ Bone complications in SCA — ranging from bone pain crises and infarction to avascular necrosis (AVN), osteopenia, and fragility fractures — are common, clinically consequential, and increasingly prevalent as more patients survive into adulthood. These features make accurate, early detection of impaired osteoblast function and abnormal bone formation a priority for clinicians and researchers working in high-burden settings such as Zaria^1^,^2^.

The skeletal pathology of SCA is multifactorial. Recurrent marrow and cortical ischemia from vaso-occlusion, marrow hyperplasia, chronic inflammation, oxidative stress, and endocrine disturbances interact to disrupt the balance between osteoclastic resorption and osteoblastic formation. These pathophysiologic processes alter the RANKL/OPG axis and other local regulators of bone remodelling, producing net bone loss, defective mineralization, and focal bone infarction that underlie clinical entities such as AVN and low bone mass^3^. A mechanistic appreciation of these pathways has motivated interest in biochemical osteoblastic markers as non-invasive indicators of dynamic skeletal activity in haemoglobinopathies^3^.

Osteoblastic markers are biochemical indices that reflect bone formation and osteoblast function. The two most widely studied are osteocalcin (OC) and bone-specific alkaline phosphatase (BALP). Osteocalcin is a vitamin-K-dependent, noncollagenous protein secreted by osteoblasts during bone matrix formation, and its serum concentration correlates with the rate of new bone synthesis. BALP, an isoform of alkaline phosphatase expressed by osteoblasts, plays a crucial role in matrix mineralization and reflects osteoblast differentiation and activity. Both markers are attractive for SCA research because they can be measured in serum, respond rapidly to changes in bone metabolism, and provide complementary information to clinical evaluation in settings where advanced imaging, such as Dual X-ray Absorptiometry (DXA) is not readily available^4^.

Empirical studies of osteoblastic markers in SCA have demonstrated altered profiles but with heterogeneous findings. Some cohorts report reduced osteocalcin and BALP, suggesting impaired osteoblast function and low bone formation, while others observe variable changes depending on age, disease severity, and treatment exposures^5^. Animal models likewise link SCA genotypes to reduced Insulin-like Growth Factor 1 (IGF-1) and impaired osteoblast differentiation, strengthening the biological plausibility of impaired osteoblastic activity in SCA^6^. Recent case-control studies in North Africa have reported significantly lower serum osteocalcin in children with sickle cell disease compared to controls, supporting the diagnostic relevance of osteoblastic markers in identifying altered bone formation and its contribution to skeletal morbidity^7^.

Despite their promise, several methodological and biological challenges constrain the diagnostic translation of OC and BALP in SCA. Both markers show circadian and pre-analytical variability, are influenced by age, sex, pubertal status, vitamin D status, and endocrine milieu, and may be confounded by transfusion history and disease activity common in SCA^8^,^9^. Assay heterogeneity and the lack of SCA-specific reference ranges — particularly for African populations — further limit interpretability. Consensus statements on biochemical markers of bone formation emphasize their utility for dynamic monitoring and research but underscore the need for standardization, context-specific reference data and studies that link marker thresholds to clinically relevant outcomes such as avascular necrosis and fractures^9^.

Against this background, a focused evaluation of osteocalcin and BALP in a Nigerian SCA cohort is timely and necessary. A Zaria study designed to measure these osteoblastic markers and to describe their diagnostic relevance in SCA would address an important operational question: can osteocalcin and BALP serve as reliable, affordable indicators of osteoblast activity and early skeletal dysfunction in Nigerian patients with SCA? Establishing locally relevant values and understanding their clinical significance will help clinicians identify patients at higher risk of skeletal morbidity and guide preventive interventions such as vitamin D supplementation and optimized supportive care — ultimately aiming to reduce bone-related complications in a population with one of the highest global burdens of SCA^1^,^2^.

## Materials and methods

This study was a hospital-based, cross-sectional comparative investigation carried out in the Departments of Haematology and Chemical Pathology, Ahmadu Bello University Teaching Hospital (ABUTH), Shika-Zaria, Kaduna State, Nigeria. Zaria, situated at a latitude of 11°02′31″N and a longitude of 7°42′E, has an estimated population of about 800,000 and lies within a tropical climate with distinct dry and rainy seasons. The prevalence of sickle cell anaemia (SCA) in this region is influenced by environmental and socio-cultural factors, including poverty, harsh climatic conditions, and the practice of consanguineous marriages.

A total of 180 participants were enrolled through consecutive sampling. The study population comprised 60 sickle cell anaemia (SCA) patients in bone pain crisis (BPC), 60 SCA patients in steady state, and 60 apparently healthy, age- and sex-matched HbAA controls. SCA participants were recruited from the Haematology Daycare Unit, Accident and Emergency Department, and Haematology Outpatient Clinic of ABUTH, while controls were drawn from hospital staff, students, and relatives.

Bone pain crisis was defined as a sudden episode of varying severe pain in patients with sickle cell disease, resulting from occlusion of the small blood vessels supplying the bone marrow, in the absence of trauma or any other identifiable pathology^10^. Steady state was defined as the absence of pain, blood transfusion, infection, or hospital admission within the four weeks preceding recruitment^11^. Eligible participants for the three study groups were adults (≥18 years) with a confirmed haemoglobin genotype. The inclusion criteria comprised: (i) HbSS individuals either in vaso-occlusive crisis or steady state, and (ii) healthy HbAA controls. Exclusion criteria were: (i) estimated glomerular filtration rate (eGFR) ≤60 mL/min/1.73 m^2^, calculated using the CKD-EPI formula, (ii) presence of significant bone deformities, and (iii) medical conditions or ongoing therapies known to affect bone metabolism.

The sample size was determined using the power formula for mean comparison at 90% power and a 5% significance level, yielding a minimum of 46.6 participants per group. After adjusting for a 28% non-response rate, 60 participants were recruited per group. Ethical approval was granted by the ABUTH Health Research Ethics Committee (Ref: ABUTHZ/HREC/F43/2023), and written informed consent was obtained from all participants.

Data collection comprised interviewer-administered questionnaires, physical examination, and biospecimen sampling. Venous blood (5 mL) was obtained under aseptic conditions and processed within one hour. Serum was separated by centrifugation at 3000 rpm for 5–10 minutes and stored at −80 °C until analysis. Participants also provided spot urine samples, which were stored alongside the serum specimens.

Biochemical analyses were carried out at the Chemical Pathology Laboratory, ABUTH, using standard methods. Osteocalcin and BALP, markers of bone formation, were measured by a sandwich ELISA. Absorbance readings for both assays were obtained using Rayto Microplate Reader at 450 nm, and concentrations were derived using standard curves. Quality control was ensured using commercial calibrators and control sera, with intra- and inter-assay CVs <10%.

Data were analyzed using Microsoft Excel and SPSS version 25.0 for Windows (SPSS Inc., Chicago, IL, USA). Qualitative variables were presented as frequencies, percentages, charts, and tables. The distribution of quantitative variables was assessed using the Kolmogorov-Smirnov test. Normally distributed data were expressed as mean ± standard deviation, while non-normally distributed data were summarized as median with interquartile range. Comparisons of serum osteocalcin and BALP concentrations across the three study groups were performed using the Kruskal-Wallis test, while post-hoc analysis was done using Dunn's Test. Associations between osteocalcin and BALP were evaluated with Spearman's correlation. Receiver Operating Characteristic (ROC) curves were generated to assess the diagnostic performance of the biochemical markers. Statistical significance was set at p ≤ 0.05.

## Result

The mean ages across groups were similar (approximately 25 years), with no significant difference (p = 0.878). Gender distribution was identical across groups, with 40% males and 60% females. Most participants were single in all groups, though a slightly higher proportion of HbAA controls were married (23.3%) compared to SCA groups. Regarding education, tertiary education was most common among controls (53.3%), whereas SCA patients had a higher proportion with secondary education (61.7%). Body mass index (BMI) differed significantly between groups (p < 0.001). HbAA controls had a higher mean BMI (23.34 ± 4.75) compared to both SCA crisis (20.01 ± 2.34) and steady state groups (20.07 ± 2.04). Underweight prevalence was highest in SCA groups (21.7% each), while overweight and obesity were more represented among controls. – Table I.

**Table I:** Clinico-Socio-demographic characteristics of the study participants

Variable	SCA in Crisis n = 60	SCA in Steady Staten = 60	HbAA n = 60	p-value
**Age (Mean±SD)**	25.3 ± 7.87	24.7 ± 7.21	24.83±4.83	0.878

**Gender**				
Male	24 (40.0)	24 (40.0)	24 (40.0)	
Female	36 (60.0)	36 (60.0)	36 (60.0)	

**Marital status**				
Married	11 (18.3)	9 (15.0)	14 (23.3)	
Single	49 (81.7)	51 (85.0)	46 (76.7)	

**Highest level of education**				
None	2 (3.3)	0 (0.0)	0 (0.0)	
Primary	6 (10.0)	4 (6.7)	0 (0.0)	
Secondary	37 (61.7)	37 (61.6)	28 (46.7)	
Tertiary	15 (25.0)	19 (31.7)	32 (53.3)	

**BMI (mean±SD)**	20.01 ±2.34	20.07 ± 2.04	23.34 ±4.75	**<0.001**

**BMI Group**				
Underweight	13 (21.7%)	13 (21.7%)	7 (11.7%)	
Normal weight	44 (73.3%)	46 (76.7%)	35 (58.3%)	
Pre-obesity	3 (5.0%)	1 (1.6%)	14 (23.3%)	
Obesity	0(0.0%)	0(0.0%)	4(6.7%)	

Median serum osteocalcin levels were highest in SCA patients in crisis (26.53 ng/mL), slightly lower in those in steady state (24.40 ng/mL), and lowest in HbAA controls (16.07 ng/mL), with the differences reaching statistical significance (p = 0.022). Similarly, serum bone alkaline phosphatase (BALP) was highest in SCA crisis patients (23.51 µg/l), intermediate in those in steady state (14.42 µg/l), and lowest in controls (9.95 µg/l), with highly significant differences across groups (p < 0.001). - Table IIa.

**Table IIa:** Serum levels of Osteocalcin and Bone Alkaline Phosphatase among the study populations

Variable	SCA in Crisis	SCA in Steady State	HbAA	*p* value
		
		Median (IQR)		
**Serum osteocalcin (ng/ml)**	26.53 (25.83)	24.40 (26.21)	16.07 (20.83)	**0.022**
**Serum BALP (µg/l)**	23.51 (56.88)	14.42 (35.11)	9.95 (11.2)	**<0.001**

**NB:** Kruskal-Wallis non-parametric test (the data is not normally distributed)

Pairwise comparisons showed that osteocalcin levels were significantly higher in SCA patients in crisis compared to HbAA controls (p < 0.001), though the difference between crisis and steady state was not statistically significant (p = 0.214). The BALP level in SCA in crisis was significantly higher than both steady state (p = 0.040) and controls (p < 0.001). Steady state levels were also significantly elevated compared to controls (p < 0.001). – Table IIb.

**Table IIb:** Post hoc analysis of Serum levels of Osteocalcin and Bone Alkaline Phosphatase among the study populations

Study groups	Osteocalcin (ng/ml)	BALP(µg/l)
**HbSS in crisis vs HbSS in steady state**	0.214	**0.040**
**HbSS in crisis vs HbAA controls**	**<0.001**	**<0.001**
**HbSS in steady state vs HbAA controls**	0.068	**<0.001**

Age-related analysis revealed a decline in both osteocalcin and BALP with increasing age, though the differences were not statistically significant (p = 0.233 and p = 0.554, respectively). Gender did not significantly influence either marker, with comparable levels between males and females. However, BMI showed significant associations with osteocalcin (p = 0.020). Overweight individuals had the highest median osteocalcin (59.37 ng/ml), while underweight participants had the lowest (21.19 ng/ml). BALP levels varied widely across BMI classes but did not show significant differences (p = 0.424).. – Table III

**Table III:** Comparison of Serum levels of Osteocalcin and Bone Alkaline Phosphatase among participants by age grouping

Variables	Serum osteocalcin Median (IQR) ng/ml	Serum BALP Median (IQR) µg/l
**Age groups**		
=20 (n=14)	33.15 (35.37)	34.05 (55.97)
21-34 (n=37)	26.93 (25.86)	23.04 (80.05)
=35 (n=9)	22.12 (25.46)	19.0 (52.57)
*p* value	0.233	0.554

**Gender**		
Female (n=36)	28.96 (26.47)	25.09 (51.26)
Male (n=24)	26.02 (24.79)	21.39(32.38)
*p* value	0.916	0.786

**BMI Classes**		
Underweight (n=13)	21.19 (11.80)	23.78 (97.6)
Normal weight (n=44)	28.76 (26.07)	25.58 (68.52)
Overweight (n=3)	59.37 (35.37)	18.31 (52.57)
Obese (0)	-	-
**p-value**	**0.020**	0.424

Kruskal Wallis Test for non-parametric variables was used

The scatterplot demonstrated a weak positive correlation (r = 0.166) between serum osteocalcin and BALP levels in patients with SCA in crisis. However, this relationship did not reach statistical significance (p = 0.206).. – Figure I.

**Figure I:**
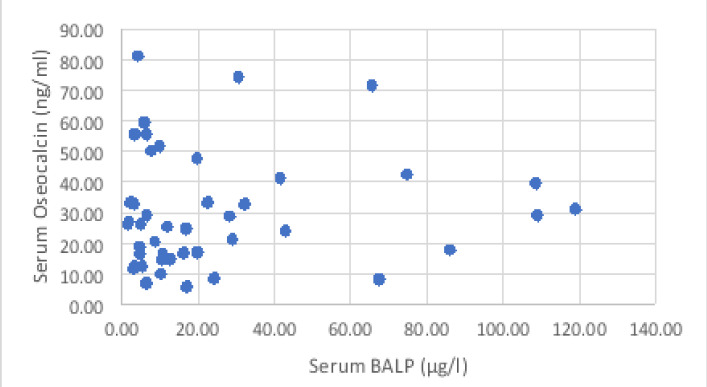
Correlation between serum osteocalcin with serum Bone Alkaline Phosphatase in SCA patients in crisis

The ROC analysis compared the diagnostic accuracy of bone alkaline phosphatase (BALP) and osteocalcin in differentiating sickle cell anaemia states from controls. Serum BALP showed superior diagnostic performance with an area under the curve (AUC) of 0.789, indicating good discriminatory ability. At a criterion of >3.36 µg/L, BALP achieved a sensitivity of 98.3% but a modest specificity of 50.0%, suggesting it is highly effective in detecting affected individuals but less precise in ruling out false positives. In contrast, serum osteocalcin had a lower AUC of 0.592, reflecting only fair diagnostic value. Using a criterion of >23.58 ng/mL, osteocalcin demonstrated 61.7% sensitivity and 60.0% specificity, which are considerably weaker than those of BALP..

## Discussion

This study demonstrates significantly elevated serum concentrations of osteocalcin (OC) and bone-specific alkaline phosphatase (BALP) in Nigerian adults with sickle cell anaemia (SCA), particularly during bone pain crises (BPC), compared to steady-state patients and healthy HbAA controls. These findings underscore the potential utility of these osteoblastic markers as indicators of heightened bone turnover in response to the complex skeletal pathology of SCA, including recurrent ischemia, chronic inflammation, and marrow hyperplasia^1^,^2^,^12^. The observed elevations align with the pathophysiology of SCA, where compensatory osteoblastic activity attempts to counterbalance increased bone resorption and remodelling disruptions^3^,^13^.

Demographic characteristics revealed comparable mean ages across groups (approximately 25 years) and a female predominance (60%), reflecting the typical patient profile in a tertiary care setting like Zaria. Notably, SCA patients exhibited lower body mass index (BMI) (approximately 20 kg/m^2^) compared to controls (23.34 kg/m^2^), with a higher prevalence of underweight status (21.7%). This nutritional disparity, potentially linked to deficiencies in vitamin D and zinc prevalent in SCA, may exacerbate skeletal morbidity^14^,^15^. Significant associations between OC levels and BMI categories (highest in overweight individuals at 59.37 ng/mL, lowest in underweight at 21.19 ng/mL) suggest OC's sensitivity to metabolic and nutritional influences, whereas BALP showed no such variation, indicating greater specificity for osteoblastic activity^4^. These findings are consistent with reports from other SCD cohorts, where young adult patients also exhibited lower BMI and a high prevalence of underweight status, reflecting chronic haemolysis and increased metabolic demands^16^,^17^. Previous studies similarly demonstrated that OC levels are influenced by nutritional status and body composition in SCD patients, with higher levels in better-nourished individuals, whereas BALP remains largely independent of BMI and more reflective of intrinsic osteoblast activity^7^,^18^. Collectively, these observations reinforce the impact of nutritional and metabolic factors on bone formation markers in SCA, highlighting the importance of monitoring body composition and micronutrient status to mitigate skeletal morbidity.

Serum OC and BALP levels were significantly elevated in SCA, with the highest values during BPC (OC: 26.53 ng/mL; BALP: 23.51 µg/L), followed by steady state (OC: 24.40 ng/mL; BALP: 14.42 µg/L), and lowest in controls (OC: 16.07 ng/mL; BALP: 9.95 µg/L). These differences were statistically significant (p=0.022 for OC, p<0.001 for BALP), suggesting increased osteoblastic activity during acute crises, likely driven by vaso-occlusion-induced infarction and inflammatory dysregulation of the RANKL/OPG axis^3^,^19^. Pairwise comparisons confirmed BALP's superior discriminatory ability across all groups, while OC significantly differentiated BPC from controls but not BPC from steady state. These patterns are consistent with prior studies reporting elevated bone turnover markers in SCA as a response to chronic haemolysis and oxidative stress^20^,^21^. However, contrasting findings in paediatric North African cohorts, which reported lower OC levels, may reflect age-related differences, pubertal influences, or variations in disease severity.^7^ Studies from India and Saudi Arabia further suggest that genetic factors, such as high-HbF phenotypes, or iron overload, may modulate marker profiles^22^,^23^.

Age and gender analyses showed non-significant declines in OC and BALP with increasing age (p=0.233 and p=0.554, respectively) and no gender differences, consistent with post-pubertal stabilization of bone turnover.^24^ The weak positive correlation between OC and BALP in BPC (r=0.166, p=0.206) indicates complementary roles, with BALP reflecting matrix mineralization and OC indicating bone matrix synthesis^4^,^25^. These findings were similar to findings from several studies, notably that of Sarrai et al.^26^ and Fung et al.^27^ However, some SCD cohorts have reported stronger coupling between OC and BALP during periods of increased bone turnover. For example, Kandil et al.^28^ demonstrated that during BPC and acute complications, bone markers may show moderate correlations, reflecting synchronized but pathologically accelerated bone turnover.

Receiver Operating Characteristic (ROC) analysis established BALP's superior diagnostic performance (AUC=0.789, sensitivity=98.3%, specificity=50.0% at >3.36 µg/L) compared to OC (AUC=0.592, sensitivity=61.7%, specificity=60.0% at >23.58 ng/mL), with a significant difference in diagnostic ability (p=0.0004). This positions BALP as a more reliable marker for detecting SCA-related bone metabolic alterations, particularly in resource-constrained settings where advanced imaging modalities like DXA are limited^29^,^30^.

The observed elevation of OC and BALP underscores their promise as affordable, non-invasive markers for monitoring skeletal impairment in SCA, with potential to guide preventive interventions such as vitamin D supplementation or hydroxyurea use to reduce risks like avascular necrosis^31^,^32^. Emerging data also indicate that therapies aimed at vascular remodelling, or addressing hydroxyurea's influence on bone metabolism, may provide additional insights for patient care^33^,^34^. Nonetheless, the cross-sectional study design restricts causal interpretation, and factors such as transfusion history or circadian fluctuations could influence marker levels, although standardized sampling helped minimize these effects^8^,^9^. To strengthen clinical application, future longitudinal, multi-center studies should combine biochemical markers with imaging and establish context-specific reference intervals, thereby linking cut-off values to outcomes such as fractures and osteonecrosis. Also, circadian variations of OC and BALP can be minimized in future research by collecting fasting blood samples at a standardized early-morning time, controlling pre-collection activity, and supplements.

## Conclusion

Elevated serum OC and BALP levels in SCA, particularly during BPC, reflect intensified osteoblastic activity, with BALP demonstrating superior diagnostic performance. Routine integration of these markers into SCA management could facilitate early identification of skeletal complications in high-burden settings like Nigeria, improving patient outcomes through targeted interventions.

## Figures and Tables

**Figure II:**
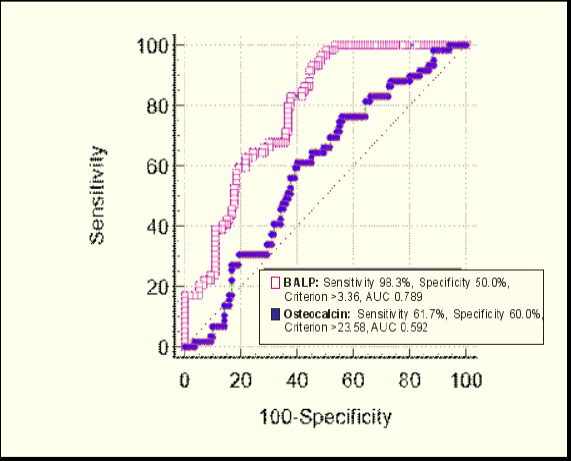
Comparison of ROC curves of Serum BALP and Serum Osteocalcin
